# The Interplay between the DNA Damage Response (DDR) Network and the Mitogen-Activated Protein Kinase (MAPK) Signaling Pathway in Multiple Myeloma

**DOI:** 10.3390/ijms25136991

**Published:** 2024-06-26

**Authors:** Panagiotis Malamos, Christina Papanikolaou, Maria Gavriatopoulou, Meletios A. Dimopoulos, Evangelos Terpos, Vassilis L. Souliotis

**Affiliations:** 1 Institute of Chemical Biology, National Hellenic Research Foundation, 116 35 Athens, Greece; pmalamos@eie.gr (P.M.); chrpapa@eie.gr (C.P.); 2 Department of Clinical Therapeutics, School of Medicine, National and Kapodistrian University of Athens, 115 28 Athens, Greece; mgavria@med.uoa.gr (M.G.); mdimop@med.uoa.gr (M.A.D.); eterpos@med.uoa.gr (E.T.)

**Keywords:** multiple myeloma (MM), DNA damage response (DDR), mitogen-activated protein kinase (MAPK), DDR/MAPK interplay, combination therapy

## Abstract

The DNA damage response (DDR) network and the mitogen-activated protein kinase (MAPK) signaling pathway are crucial mechanisms for the survival of all living beings. An accumulating body of evidence suggests that there is crosstalk between these two systems, thus favoring the appropriate functioning of multi-cellular organisms. On the other hand, aberrations within these mechanisms are thought to play a vital role in the onset and progression of several diseases, including cancer, as well as in the emergence of drug resistance. Here, we provide an overview of the current knowledge regarding alterations in the DDR machinery and the MAPK signaling pathway as well as abnormalities in the DDR/MAPK functional crosstalk in multiple myeloma, the second most common hematologic malignancy. We also present the latest advances in the development of anti-myeloma drugs targeting crucial DDR- and MAPK-associated molecular components. These data could potentially be exploited to discover new therapeutic targets and effective biomarkers as well as for the design of novel clinical trials. Interestingly, they might provide a new approach to increase the efficacy of anti-myeloma therapy by combining drugs targeting the DDR network and the MAPK signaling pathway.

## 1. Introduction

Multiple myeloma (MM) is a hematologic malignancy characterized by the overproduction of monoclonal immunoglobulins and the clonal proliferation of long-lived plasma cells [1,2]. Monoclonal gammopathy of undetermined significance (MGUS) is a premalignant condition, which may progress to MM [3,4]. Three to four percent of people over the age of fifty have MGUS. Recent data suggest that chronic antigenic stimulation, black race, older age, male sex, diabetes, certain pesticides, family history, inflammatory conditions, and obesity are risk factors for developing MGUS [3,5,6,7]. Moreover, smoldering MM (SMM), an intermediate more advanced premalignant stage, is clinically identified in certain patients [8,9]. Annually, ~1% of MGUS cases progress to SMM; for those with SMM, the relevant risk of developing MM is much higher: ~10% per year for the first five years and 3% per year for the subsequent five [10,11].

Treatment of MM is a multifaceted approach that depends on various factors, including the stage of the disease, the patient’s overall health, and individualized treatment goals [12,13,14]. It involves combinations of drugs with several mechanisms of action, such as corticosteroids, including dexamethasone and prednisolone [15]; alkylating agents, namely melphalan [16] and cyclophosphamide [17]; histone deacetylase inhibitors (HDACi; panobinostat) [18]; anthracyclines, for instance doxorubicin [19]; proteasome inhibitors (PIs; bortezomib, carfilzomib and ixazomib) [20]; immunomodulatory drugs (IMID; thalidomide, lenalidomide and pomalidomide) [21]; high-dose chemotherapy followed by autologous stem cell transplantation [22]; monoclonal antibodies (mAbs; elotuzumab, daratumumab, isatuximab) [23]; chimeric antigen receptor (CAR) T-cell therapy; etc. [24,25,26]. Despite these advancements, MM remains an incurable disease, and the need for new treatment strategies is mandatory.

Using modern biology technologies, molecular characteristics of MGUS and SMM as well as the progression to active MM are now better understood [27]. Interestingly, several reports identified mutations in genes involved in the DNA damage response (DDR) network and the mitogen-activated protein kinase (MAPK) system [28]. Therefore, in this review, we provide data from the current literature regarding aberrations in the DDR network, the MAPK system, and their interplay that are involved in the onset and progression of MM and the development of drug resistance. The latest advances in anti-myeloma drugs targeting critical DDR- and MAPK-related components are also elucidated.

## 2. The DDR Network

**Figure 1:**
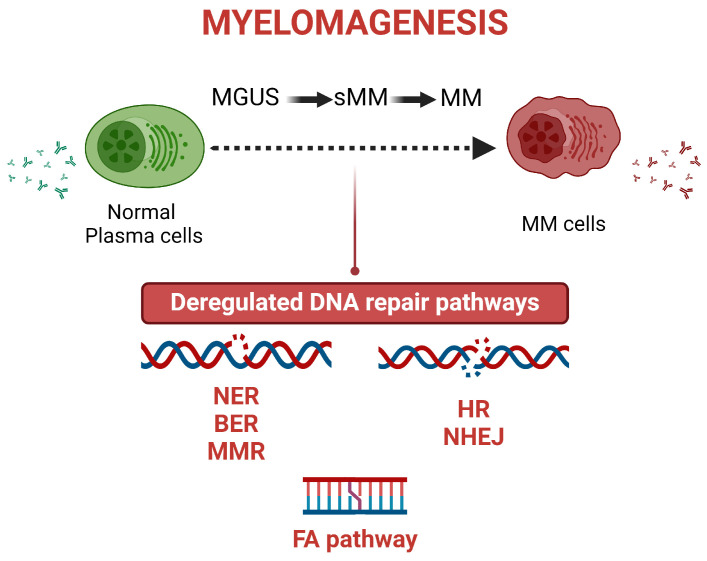
Deregulated DNA repair pathways involved in myelomagenesis. “BioRender.com (accessed on 18 June 2024)”.

Damage to DNA occurs due to external [ultraviolet (UV) and ionizing radiation, genotoxic drugs] or internal factors (oxidative stress, telomere erosion, replication fork collapse) [29]. To overcome these alterations in the chemical structure of DNA, cells have developed a complex system of pathways, called the DDR network, that recognize and resolve the damage, thus protecting the integrity of the genome [30]. DDR is triggered following the detection of a DNA lesion. Next, a signal transduction cascade is activated and results in the stimulation of sophisticated mechanisms for genome protection, including DNA repair pathways, cell cycle checkpoints, and apoptosis. On the other hand, deregulated DDR may result in mutagenesis and genomic instability [31]. Given that DDR regulates the cellular decision to remove the DNA damage or to activate apoptosis, it is involved in the onset and progression of several diseases, including MM (Figure 1), as well as in the response to therapeutic interventions.

### 2.1. Deregulation of the DDR Network in the Onset and Progression of MM

Previous studies have shown that DNA repair mechanisms are altered in MM (Table 1). In fact, deregulation in the Base Excision Repair (BER) pathway plays an important role in MM progression. For example, Liao and colleagues reported that two major BER-related apurinic/apyrimidinic nucleases (APEX1 and APEX2) crosstalk with p73, a transcriptional regulator of RAD51, and results in its transcriptional upregulation, thus increasing the efficiency of homologous recombination (HR) and driving genomic instability in MM [32]. Moreover, a study of polymorphisms of BER-associated genes correlates alterations in *APEX1* with a reduction in MM patients’ overall survival [33]. In patients’ samples, both *APEX1* and *APEX2* gene expressions were increased during myelomagenesis [34]. Also, researchers found that high expressions of certain BER genes, such as *MPG* (N-methylpurine DNA glycosylase) and *PARP3* [Poly(ADP-ribose) polymerase 3], are linked to improved overall survival in MM patients who received autologous stem cell transplantation. On the other hand, increased expressions of *PARP1* and *POLD2* (DNA polymerase delta subunit 2) are associated with worse outcomes in MM, suggesting that targeting the BER pathway might improve treatment effectiveness [35,36,37].

Moreover, the gene expression patterns in normal plasma cells and newly diagnosed MM samples revealed that upregulation of the Nucleotide Excision Repair (NER) protein ERCC3 (excision repair cross-complementation group 3) is linked to poorer survival. Additionally, researchers have identified 34 NER-related genes with differential expression in MM plasma cells, along with 23 genes with copy-number variations [38]. Interestingly, polymorphisms of NER have been shown to impact treatment outcomes in MM patients undergoing autologous bone marrow transplantation [52].

It is known that the defective Mismatch Repair (MMR) mechanism results in increased mutation rates, particularly in microsatellite DNA regions. This defect, known as microsatellite instability, was observed in many MM patients and becomes more common as the disease progresses and during relapse [53]. Alterations in MMR genes (*hMSH2*, *hMLH1*, and *hPMS1*) have been identified in malignant disorders of B-cells and were associated with aggressive behavior [39,40]. Defective MMR is also implicated in drug resistance [54,55].

The homologous recombination repair (HR/R) mechanism removes DSBs that are formed following therapeutic treatment with several anti-myeloma drugs, such as topoisomerase inhibitors and DNA crosslinking agents. Previous studies have shown elevated expressions of HR/R-associated genes, namely *RAD50* and *RAD51*, and increased HR/R activity in MM cell lines and primary MM cells compared with normal plasma cells [41,47]. Since HR/R plays an important role in the recovery of the stalled replication fork and the repair of interstrand cross-links (ICLs), it is of particular importance in drug resistance of the fraction of proliferating MM cells. Indeed, previous studies have shown that following treatment of MM patients with high-dose melphalan (HDM) and autologous stem cell transplantation (ASCT), higher expressions of *BRCA1*, *PRKDC* (*DNA-PK*), and *PARP1* genes are linked to poorer outcomes [43]. Moreover, genetic variations in *PARP*, *RAD51*, *MUTYH*, *OGG1*, *PCNA*, *TPMT*, and *XPC* are associated with disease progression [42].

Studies in mice have highlighted the crucial roles of core proteins involved in Non-Homologous End Joining (NHEJ) repair mechanism in preserving genomic stability [56]. In some MM cell lines, such as RPMI-8226, NHEJ activity appears to be compromised, while it remains functional in others, including U266 and OPM2 [57]. A study on MM patients treated with thalidomide also revealed that those with specific gene polymorphisms in *ERCC1*, *ERCC5*, or *XRCC5* (*KU80*) had higher response rates with longer overall survival being associated with polymorphisms in *ERCC1* and *XRCC5*. Polymorphisms or abnormal expression of genes such as *XRCC4*, *XRCC6* (*KU70*), *DCLRE1C/Artemis*, and *DNA ligase IV* (*LIG4*) have also been linked to MM risk [44,45,47]. Indeed, increased levels of *DCLRE1C/Artemis*, *DNA–PKcs*, and *XRCC4* proteins were observed in MM cells, while elevated expressions of *XRCC5* and *DCLRE1C/Artemis* genes were linked to poorer prognosis in MM patients [46]. Previous reports have also shown that NSD2 (Nuclear Receptor Binding SET Domain Protein 2), a factor with many biological functions, including DNA repair, plays a crucial role in MM relapse and treatment resistance [48]. In line with these data, loss of NSD2 function reduces the expression of DNA repair genes like *RAD51*, *TP53BP1*, and *XRCC4* and enhances DNA damage accumulation. On the other hand, overexpression of *NSD2* increases DNA repair efficiency, which may contribute to drug resistance, particularly in t(4;14) MM cases [49]. Alternative NHEJ (alt-NHEJ) is a DNA repair pathway that is vital for genomic instability and the survival of MM cells. Higher gene expression of *LIG3* (component of alt-NHEJ; also involved in NER and BER) in MM patients is linked to shorter survival, especially in advanced disease stages. LIG3 protein levels are elevated in bortezomib-resistant compared to bortezomib-sensitive MM cells; knocking down *LIG3* increases DNA damage and inhibits MM cell growth both in vitro and in vivo [50].

Fanconi anemia (FA) is a rare chromosomal instability syndrome, which has been linked to pathogenic variations in at least 22 genes that make up the FA pathway. Interestingly, FA patients’ cells are very sensitive to ICL-inducing drugs, suggesting that FA pathway is implicated in the repair of ICLs [58]. In line with these data, melphalan-resistant myeloma cells express high levels of *FANCF* (*FA Complementation Group F*) and *RAD51C*; depletion of *FANCF* helps overcome resistance [51].

Gene expression analyses of MM patients treated with HDM and ASCT have revealed the prognostic significance of genes involved in several DNA repair pathways, including NHEJ, HR/R, FA, NER, MMR, and BER [59]. Among 84 examined genes, 22 were found to have prognostic value for both event-free and overall survival. These genes included five related to NHEJ [three with negative (*NSD2*, *RIF1*, *XRCC5/KU80*) and two with positive prognostic value (*PNKP* and *POLL*)], six to HR/R [five with negative (*EXO1*, *BLM*, *RPA3*, *RAD51*, *MRE11*) and one with positive prognostic value (*ATM*)], three related to FA [all with negative prognostic value (*RMI1*, *FANCI* and *FANCA*)], eight to NER [six with negative (*PCNA*, *RPA3*, *LIG3*, *POLD3*, *ERCC4*, *POLD1*) and two with positive prognostic value (*ERCC1*, *ERCC5*)], two involved in MMR [both with negative prognostic value (*EXO1* and *MSH2*)], and one in BER with negative prognostic value (*LIG3*).

One of the most important independent prognostic factors associated with poor clinical outcome in MM is the deletion of the short arm of chromosome 17 (del(17p)), where the tumor suppressor *TP53* gene is located [60,61,62]. It is widely accepted that deregulation of the *TP53* gene is important in the onset of several types of cancer, including MM. Three subtypes of deregulated *TP53* are found in newly diagnosed MM patients: monoallelic deletion as part of the del(17p) (8%), monoallelic mutations (6%), and biallelic inactivation of *TP53*, that is a deletion and a mutation, known as Double Hit MM (4%) [62,63,64]. While it is not clear how monoallelic mutations affect prognosis, biallelic and del(17p) patients seem to have the worst prognosis [65].

### 2.2. The DDR Network in the Outcome of Anti-Myeloma Therapy

Extensive observations suggest that the DDR network is implicated in the outcome of genotoxic therapy. Indeed, studies have shown that in vitro resistance to the nitrogen mustard melphalan [16] is linked to increased efficiency of DNA repair mechanisms, including ICL repair [66] and FA/BRCA pathway [51]. In order to elucidate the role of DDR in the outcome of melphalan-treated patients, previous studies reported the formation and repair of DNA damage in peripheral blood mononuclear cells (PBMCs) and bone marrow plasma cells (BMPCs) following in vivo (therapeutic) or ex vivo melphalan treatment [67,68,69,70]. The authors reported that ΜΜ patients, responders to melphalan therapy, are characterized by lower DNA repair capacity and higher accumulation of melphalan-induced DNA damage than non-responders, suggesting that quantification of drug-induced DNA damage formation/repair may help in the selection of patients who may profit from melphalan therapy. Interestingly, they reported that DSB repair (DSB/R) inhibitors, such as the NHEJ inhibitor SCR7, significantly enhanced the cytotoxicity of melphalan against malignant plasma cells, suggesting a promising strategy for the treatment of MM [69].

DSB/R inhibitors are not the only DDR modifiers used in MM therapy. Indeed, previous studies have shown that the combined treatment with inhibitors of ATM (KU-55933) and ATR (VE-821) seriously reduced survival of MM cell lines that exhibited high levels of endogenous DNA damage [71]. Also, PIM-2, a serine/threonine kinase that interacts with DDR and plays a critical role in promoting cell survival and preventing apoptosis, is commonly found upregulated in MM [72,73]. Another study has shown that LT-171-861, a synthetic new PIM-2 inhibitor, induced DNA damage by inhibiting the HR/R pathway, activated apoptosis in MM cells, and suppressed tumor growth in MM xenograft models [74]. Moreover, the PARP inhibitor olaparib could amplify the anticancer effect of LT-171-861 by reducing the growth of tumors in MM xenografted nude mice.

Panobinostat is an HDACi, which blocks cell cycle progression, induces apoptosome formation, and down-regulates the anti-apoptotic *Bcl-2* gene [75]. Panobinostat showed synergistic anti-MM effects when combined with genotoxic drugs in vitro [76]. Indeed, in a recent report, the authors studied the biological effects of the ex vivo co-treatment of panobinostat and melphalan in BMPCs and PBMCs from MM patients [77]. They found that this combination treatment reduced the efficiency of critical DNA repair mechanisms (NER and DSB/R), augmented the accumulation of cytotoxic DSB lesions, and induced apoptosis in BMPCs but not in PBMCs from the same patients or healthy controls. These data suggest that, compared with melphalan alone, the combined treatment of melphalan and panobinostat showed increased anti-myeloma efficacy and lower side effects.

## 3. The MAPK System

The MAPK signaling pathways organize a network that is highly interconnected and regulates many cellular processes, including cell growth, differentiation, inflammation, cell stress response, proliferation, metabolism, migration, and apoptosis [78]. MAPK is a very complicated network. Indeed, 24 MAP3Ks regulate 7 MAP2Ks, which in turn regulate 14 MAPKs, making up the MAPK signaling network [79]. In mammals, between the 14 MAPKs, three signaling pathways, namely MEK/ERK, c-Jun N-terminal kinase (JNK), and p38, are determined by their genome homology, functional redundancy, and shared activation mechanisms [78,80,81,82]. Interestingly, disruption of the MAPK signaling cascade plays a role in the pathogenesis of different human cancer types [81,83,84].

### 3.1. MAPK Signaling Pathways and Myelomagenesis

Using the next-generation sequencing (NGS) technology, a deeper understanding of the molecular characteristics of MGUS/SMM and the progression toward active MM has become possible [85]. Numerous mutations were discovered by whole-exome sequencing (WES), with MAPK signaling being one of the most commonly affected pathways. Several reports identified that the *NRAS*, *KRAS*, and *BRAF* mutation frequency rises from 24% in SMM [86] to 50% in newly diagnosed MM cases [87,88,89,90,91] and up to 80% in relapsed/refractory MM (RRMM) patients [92,93].

RAS proteins belong to a family of GTP hydrolases (GTPases) that are frequently mutated in human malignancies [94,95]. There are three major isoforms of the *RAS* gene (*NRAS*, *KRAS*, and *HRAS*) that have a significant role in cell proliferation, survival, and differentiation. Although in human cancers, all three isoforms are commonly mutated oncogenes, most tumors have mutations in the *KRAS* gene [95]. In MM patients, the mutation incidence is 22–25% for *KRAS* and 20–25% for *NRAS*. The most common hotspot mutations are in the codons 12, 13, and 61 of the *NRAS* and *KRAS* genes; Q61 mutations (i.e., substitutions of glutamine at amino acid 61 by another amino acid) for *NRAS* gene are also common in MM [96]. These mutations mostly affect the activity of *NRAS* and are linked to a more aggressive phenotype and shorter overall survival [97]. Accordingly, in relapsed MM, *NRAS* mutations are linked to decreased susceptibility to the proteasome inhibitor bortezomib [98].

The development of several cancer types is also associated with mutations in the *BRAF* proto-oncogene. Among the most frequent mutations observed in the *BRAF* gene are those encoding the V600E mutant, which results in continuous activation and signal transduction, irrespective of external stimuli. Consequently, cell proliferation and invasion are increased in cancer patients harboring such mutations [99]. The V600E mutation has been specifically associated with melanoma, metastatic colorectal cancer, MM, and several other cancer types [100]. The prevalence of the *BRAF* V600E mutation is high among patients diagnosed with MM, with a frequency ranging from 2% to 4% in newly diagnosed cases and rising to approximately 8% in patients with relapsed/refractory disease or extramedullary involvement [97]. A study of 223 newly diagnosed MM patients exploring gene expression profiles and clinical data detected *BRAF* mutations in 11% of patients with an unfavorable prognosis [101]. The authors detected both *BRAF* V600E and non-V600E *BRAF* mutations, 58% of which were hypoactive or kinase inactive. It is worth noting that 44% of the hypoactive/kinase inactive BRAF patients displayed concurrent mutations in *NRAS* or *KRAS*, indicating their involvement in the pathogenesis of the disease by promoting the activation of MAPK via upstream mutated elements.

### 3.2. MM Therapy: Targeting MAPK Signaling Pathways

Since the MAPK pathway is mutated in many cancer types, including MM, it is considered to be a major therapeutic target (Table 2) [102,103]. In fact, the FDA has approved four MEK1/2 inhibitors, namely, binimetinib, trametinib, cobimetinib, and selumetinib [104], and three BRAF inhibitors (vemurafenib, dabrafenib, and encorafenib) for the treatment of several malignancies [102,103].

MEK inhibitors monotherapy in MM has shown mixed results. Indeed, the inhibition of MEK with selumetinib in MM showed a low response in relapsed/refractory MM [105]. On the other hand, trametinib had better response rates in MM patients with MAPK activation [103]. Also, a clinical trial (NCT03312530) evaluating the safety and the efficacy of cobimetinib showed promising results when this MEK inhibitor was administrated alone or in combination with venetoclax (BCL-2 inhibitor) with or without atezolizumab (PD-L1 inhibitor) in t(11;14) MM patients [106,107].

Concerning BRAF inhibition, Andrulis and colleagues examined the mutation status of *BRAF* V600E in primary tumor samples from 379 MM patients and correlated it with disease outcome [108]. They found that the presence of the *BRAF* V600E mutation was linked to the development of aggressive extramedullary diseases and shorter overall survival. Moreover, they presented a case study of an MM patient diagnosed with a *BRAF* V600E mutation and experiencing a relapse of myeloma accompanied by widespread extramedullary involvement; this patient exhibited a rapid and sustained positive response to low doses of vemurafenib.

While most cancer patients show favorable initial responses to BRAF inhibitors, resistance occurs once the ERK pathway is reactivated. To overcome this problem, combinational therapies including BRAF and MEK inhibitors or the use of new second-generation multiple inhibitors, such as the pan-RAF inhibitor tovorafenib (TAK-580; an inhibitor of wildtype BRAF, BRAF V600E, and CRAF) and avutometinib (RO-5126766, also known as CH-5126766; a dual inhibitor of Raf and MEK), were developed [119,120]. Indeed, combined regimes of cobimetinib and vemurafenib showed promising anti-MM results in advanced RRMM [109,110]. Moreover, in a phase II clinical trial (NCT02834364), combined treatment with encorafenib and binimetinib showed positive results in RRMM patients carrying a *BRAF* V600E mutation [111]. Also, Guo and colleagues reported that the RAF–MEK inhibitor RO-5126766 had antitumor activity against several solid tumors and MM with RAF–RAS–MEK pathway mutations [113]. Interestingly, in a GMMG-BIRMA phase II study (NCT02834364), the combination of binimetinib and encorafenib in RRMM patients with a *BRAF* V600E or a *BRAF* V600K mutation showed an 82% overall response rate with 9 out of 11 MM patients having at least partial response [111,112]. In addition, in vitro combination treatment of sorafenib (a RAF and VEGF2 inhibitor) and rapamycin (a potent immunosuppressive drug) showed improved results [114]. Also, a phase I clinical trial using a combination treatment of sorafenib and bortezomib found promising results in several malignancies [115]. However, when investigated in a phase II clinical trial for metastatic or unresectable renal cell carcinoma, the response rates and the progression-free survival were similar to sorafenib monotherapy [121]. In another small study on MM patients, a partial response and a continuous stable disease were observed in 2/11 individuals after sorafenib treatment [116].

The use of p38 MAPK inhibitors, such as talmapimod, plitidepsin, and ralimetinib (LY2228820), has shown good preclinical efficacy. Indeed, results from a phase II trial with talmapimod alone or in combination with bortezomib have shown encouraging response rates in the RRMM patients who had previously failed bortezomib monotherapy [117]. Moreover, plitidepsin monotherapy showed a 13% response rate in RRMM patients, and when it was combined with dexamethasone, response rates reached as high as 22% [118]. However, due to infections, short-lived clinical efficacy, and skin damage, to date, there are no FDA-approved drugs against p38 MAPK.

## 4. The DDR Network and the MAPK System Are Coordinately Activated

A series of studies have indicated that the DDR network and the MAPK signaling pathway are activated in concert. Indeed, following the activation of DDR, the MEK/ERK pathway is also activated, thus facilitating the proper induction of DDR checkpoints to arrest cell division [122]. Inhibition of the ERK/MAP kinase abrogates cell cycle checkpoint activation and results in cell proliferation in the presence of DNA lesions, thus triggering the accumulation of mutations and development of tumors [123]. On the other hand, abrogation of checkpoint activation may also induce apoptosis or cell catastrophe, thereby enhancing the efficacy of chemotherapy [124].

### 4.1. Induction of DDR Activates MAPK

**Figure 2:**
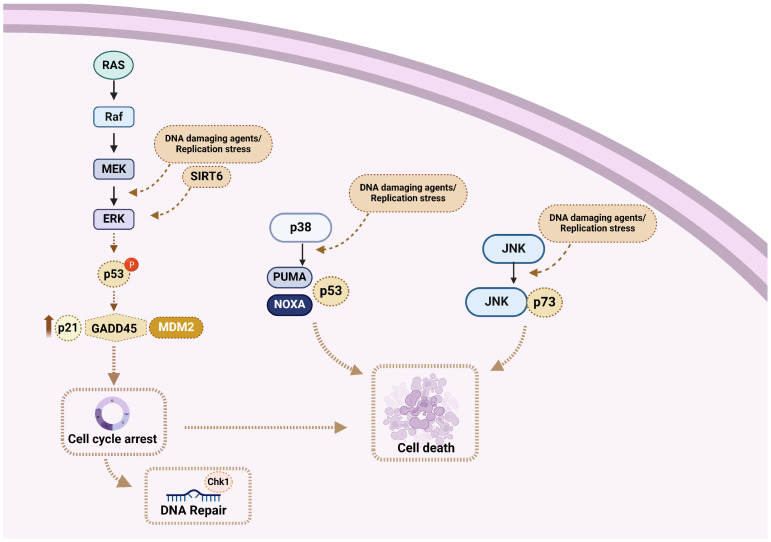
Schematic diagram displaying the interplay between the MAPK system and the DDR network. “BioRender.com (accessed on 18 June 2024)”.

Phosphorylation of the ERK/MAP kinase delivers a survival signal that counteracts pro-apoptotic effects associated with JNK and p38 MAPK activation [125,126,127]. On the other hand, Wang and colleagues found that in HeLa and A549 cell lines, the activation of ERK/MAP kinase is crucial for the induction of cisplatin-induced apoptosis [128]. Indeed, treatment of HeLa cells with cisplatin caused dose- and time-dependent activation of the MEK/ERK signaling pathway, which ultimately led to apoptosis (Figure 2). In line with these data, the pretreatment of HeLa cells with TPA (12-O-tetradecanoylphorbol-13-acetate), an activator of the ERK/MAP kinase signaling pathway, enhanced their sensitivity to cisplatin. Moreover, when HeLa cells were pretreated with an MEK1/2 inhibitor (PD98059 or U0126), cisplatin-induced apoptosis was prevented, while cisplatin-resistant HeLa cell variants showed reduced ERK phosphorylation [128]. Together, these data indicate that ERK activation is a fundamental mediator of cisplatin-induced apoptosis that functions upstream of caspase activation to start the apoptosis pathway. However, this is not a universal feature, since Chu and colleagues found that inhibition of ERK activity in PC-3 prostate cancer cells did not change their sensitivity to cisplatin [129].

Previous studies have shown that cisplatin-associated ERK/MAP kinase activation precedes p53-mediated DDR. Indeed, ERK phosphorylates p53, causing increased expression of the *p21CIP1*, *MDM2* (*mouse double minute 2 homolog*), and *GADD45* (*45kd-growth arrest and DNA damage*) genes [130]. As such, the activation of ERK may result in cell cycle arrest, providing time for the repair of cisplatin-induced DNA damage via p53. Moreover, p53 affects the sensitivity to apoptosis by activating the transcription of apoptotic genes (*BAX*) and repressing the transcription of apoptosis-inhibition genes (*BCL-2*) [131]. On the other hand, inhibition of cisplatin-induced ERK activation increases the sensitivity of cisplatin and decreases the levels of *p21CIP1*, *MDM2*, and *GADD45* [127].

Other DNA damaging factors, including etoposide, adriamycin (doxorubicin), or UV irradiation, also stimulate ERK1/2 MAP kinase in several cell lines [127,128,132]. Interestingly, in response to high or low intensity DNA insults, ERK/MAP kinase activation triggers apoptosis or cell cycle arrest, respectively [133]. Following these results, abrogation of ERK/MAP kinase activation was found when cells pretreated with MEK1/2 inhibitors were exposed to DNA damaging factors, thus verifying the role of MEK1/2 in mediating DNA damage-induced ERK activation [128]. Moreover, treatment with MEK1/2 inhibitors caused inhibition of ERK/MAP kinase and attenuation of p21CIP1 induction, resulting in the release of etoposide-induced G2/M cell cycle arrest. Furthermore, MEK1/2 inhibitors attenuated apoptosis that was induced by high doses of DNA-damaging agents. On the other hand, the excessive expression of the MEK1Q56P gain-of-function variant forced the activation of the ERK1/2 MAP kinase, making cells more susceptible to DNA damage-induced apoptosis [133]. Together, the phosphorylation and activation of the ERK/MAP kinase in the presence of DNA damage contribute to cell cycle arrest and apoptosis, thus explaining why cancer cells with high levels of ERK activation are more sensitive to DNA-damaging agents.

Interestingly, previous research showed that sirtuin 6 (SIRT6; deacetylase involved in DDR) interacts with the ERK signaling-related gene and the ERK-induced transcription factor ELK1 [134]. SIRT6 inhibits the expression of genes involved in the MAPK signaling pathway by interacting with their promoters and deacetylating H3K9 at these locations. In addition, inhibition of the ERK2/p90RSK signaling pathway induced by high SIRT6 levels increases the DNA repair by CHK1 (checkpoint kinase 1) and the resistance to DNA damage [135]. In fact, in vitro experiments and human MM xenograft models showed a relationship between SIRT6 and genomic instability of MM cells. That is, they found that MM cells have high amounts of SIRT6, which inhibit the activity of the target genes *ELK1*, *RSK2* (*ribosomal S6 kinase 2*), and *ERK2* in response to continuous DNA damage and genomic instability. The persistent DNA damage in MM causes SIRT6 to be recruited to DSBs and the downregulation of genes involved in MEK/ERK signaling. On the other hand, depletion of SIRT6 activates several ERK-related genes, including MAPK-activated *RSK2* and *ELK1*-mediated transcriptional activity, thus blocking the G2/M cell cycle checkpoint [135].

In addition to ERK/MAP kinase signaling, the JNK pathway is also activated as a result of DNA damage. Indeed, following treatment with cisplatin, DNA damage results in the stabilization and activation of p73, which creates a complex with JNK and triggers drug-induced apoptosis [136]. Moreover, several stress stimuli, including environmental stress, are mediators of cisplatin-induced apoptosis through the activation of the p38 MAPK family of signaling proteins. A previous report has shown that cisplatin causes EGFR (epidermal growth factor receptor) internalization through the phosphorylation, and thus activation, of the receptor by p38 MAPK [137]. Also, cisplatin induces stabilization of p18 Hamlet, a protein controlled by p38 MAPK, thus increasing the p53′s capacity to bind with and activate the pro-apoptotic genes *PUMA* (*p53 upregulated modulator of apoptosis*) and *PMAIP1* (*phorbol-12-myristate-13-acetate-induced protein 1*; also known as *NOXA*) [138]. Together, these results suggest that the p38 MAPK pathway plays an important role in the regulation of cisplatin-induced apoptosis.

The involvement of the JNK pathway in the response to cisplatin has been confirmed by the inhibition of the JNK that reduced cisplatin-induced apoptosis in cervical cancer cells [139]. In contrast, blocking the p38 MAPK pathway increased reactive oxygen species levels, activated the JNK pathway, and made human tumor cells more susceptible to cisplatin-induced cell death [140]. This is also consistent with another study, which found that in epithelial renal tubule cell lines, p38 MAPK inhibition increased cisplatin-induced cell death via glutathione depletion and drug transport alteration [141]. In addition, treatment of several myeloma cell lines (NCI-H929, OPM2, RPMI-8226, U266) with the bifunctional mechlorethamine derivative bendamustine causes cleavage of caspase 3 and induction of apoptosis, while all cell lines experienced G2 cell cycle arrest [142]. Interestingly, the selective p38 MAPK inhibitor SB202190 dramatically boosts bendamustine-induced apoptosis and abrogates G2/M cell cycle arrest, suggesting that the combined treatment with MAPK and DDR modifiers might be used as novel anticancer therapy.

### 4.2. Induction of MAPK Activates DDR

Progression of the cell cycle from the G0/G1 to the S phase is induced by growth factors and depends on the ERK family of MAP kinases (Figure 2). Interestingly, ERK/MAP kinase activation must be continuous to trigger S phase entry [143,144]. Immediate early genes and cyclins, among other ERK-dependent upregulated genes, are essential for promoting cell cycle progression. Therefore, growth factor-stimulated continuous ERK activation could ensure G1 phase progression by upregulating genes that promote proliferation and by downregulating genes that inhibit it. The inactivation of ERK by an MEK inhibitor or a dominant negative MEK1 at any point before the onset of the S phase decreased the S phase entry rate [145].

Prior research suggests that BRAF inhibition upregulates the expression of *p21CIP1* and *p27* and downregulates the expression of retinoblastoma protein (*pRb*), *cyclin D*, and *cyclin E* genes that are implicated in G1 cell cycle progression [146]. For example, vemurafenib promotes cell cycle arrest at the G0/G1 phase and causes apoptosis in melanoma-sensitive, but not in melanoma-resistant, cell lines harboring the *BRAF* V600E mutation [147]. Interestingly, in vemurafenib-sensitive cell lines, the combination of HDACi suberoylanilide hydroxamic acid (SAHA) with vemurafenib induced both G0/G1 arrest and apoptosis, while in vemurafenib-resistant cells, the same combination induced G0/G1 and G2/M arrest, resulting in dramatic cytostasis. It is noted that in vemurafenib-resistant cells, data from a gene expression study found MAPK hyperactivation and dysregulation of cyclins and CDKs, alterations that are consistent with the cytostatic effects of SAHA.

Even though p38 MAPK is not necessary for the DNA damage-induced G2/M checkpoint activation, it performs a crucial pro-survival role during this cell cycle arrest through the overexpression of Bcl2 family proteins. In line with these data, inhibition of p38 during G2/M checkpoint arrest results in the simultaneous reduction of Bcl2 protein levels and triggers apoptosis in a p53-independent manner [148]. Another report has shown that p38 MAPK promotes DDB2 degradation and chromatin relaxation, thus stimulating the repair of UV-induced DNA damage by the NER pathway [149]. In fact, following UV irradiation, DDB2 is recruited to the damaged DNA sites, while p38 MAPK rapidly activates and helps DDB2 ubiquitylation. Consequent degradation of DDB2 results in the recruitment of the XPC (xeroderma pigmentosum complementation group C) protein involved in the recognition of DNA damage through global genome repair (GGR), a critical subpathway of NER. Additionally, p38 MAPK helps to unfold the compacted chromatin by enhancing histone modifications, thus making UV lesions more accessible to NER factors.

CHK1, a serine/threonine protein kinase, is essential for protecting cells from stress and DNA damage during DNA replication [150]. Inhibition of this kinase causes accumulation of DNA damage, possibly due to increased replication stress. Indeed, Dai and colleagues investigated the role of the RAS/MEK/ERK pathway in relation to DNA damage in human MM cells exposed to CHK1 inhibitors and found that RAS/MEK/ERK signaling disruption significantly augmented DNA damage induced by CHK1 inhibitors and increased cells’ sensitivity [122].

Moreover, an accumulating body of evidence suggests that MAPK signaling regulates the HR/R mechanism in human cells, with JNK and ERK/MAPK pathways being positive and p38 being negative regulators of HR/R [151]. More specifically, the inhibition of MEK/ERK signaling compromised ATM activity and reduced ATM phosphorylation and localization to foci, suggesting that ERK signaling affects the formation or the stability of repair protein complexes and/or the localization of ATM required for effective HR/R. On the other hand, inhibition of ATM kinase reduced ERK phosphorylation, suggesting that ATM modulates the ERK/MAPK signaling pathway. Therefore, a regulatory feedback loop may control DDR and ERK/MAPK signaling.

Also, using siRNA screening, Köpper and colleagues revealed kinases that contribute to the increased phosphorylation of H2AX at Ser139 (γH2AX) after UV-induced replication stress [152]. They found a dramatic reduction in γH2AX after the knockdown of the *MAPK-activated protein kinase 2* (*MK2*), a kinase implicated in p38 stress signaling and G2 arrest. These results suggest that the cellular response to replication stress and the subsequent accumulation of DNA damage are directly influenced by the p38 MAPK signaling pathway [152,153].

## 5. Conclusions

Living organisms are protected against endogenous and exogenous hazards by a tightly regulated process that includes the synergistic action of the DDR network and the MAPK system. The DDR machinery, an organized network of molecular interactions, appears to play a major role in several biological pathways, including the repair of DNA lesions, the cell cycle, and cell death, with deregulation of the network resulting in mutagenesis and genomic instability. Moreover, the MAPK system regulates a variety of biological processes, such as proliferation, differentiation, inflammation, migration, metabolism, and cell survival or death. Guided by this notion, aberrations in these networks may contribute to the pathogenesis and progression of several diseases, including MM. Since these alterations may also be involved in the development of drug resistance, they might be exploited as novel therapeutic targets. Indeed, recent studies have reported that several drugs targeting the DDR network and the MAPK system are in various stages of development. It is worth noting that compared with monotherapy, combination therapy can bring several clinical benefits, including increased treatment effectiveness, decreased treatment failure rate, lower drug concentrations for each drug, fewer side effects, and lower risk of relapse. Therefore, the results presented herein potentially offer a new approach to enhance the efficacy of anti-myeloma therapy by combining DDR modulators with drugs targeting the MAPK signaling cascade.

## Figures and Tables

**Table 1:** Alterations in DDR-related genes in MM.

DDR Pathway	Gene	Alteration	Results	Ref.
BER	*RAD51*	Up	Increased HR/R efficiency and genomic instability	[32]
	*APEX1*	Up	Reduced OS, increased expression in myelomagenesis	[33,34]
	*APEX2*	Up	Increased expression in myelomagenesis	[34]
	*MPG*	Up	Increased OS in pts receiving ASCT	[35,36,37]
	*PARP3*	Up	Increased OS in pts receiving ASCT	[35,36,37]
	*PARP1*	Up	Worse outcome in MM	[35,36,37]
	*POLD2*	Up	Worse outcome in MM	[35,36,37]
NER	*ERCC3*	Up	Poorer survival in NDMM	[38]
MMR	*hMSH2*	Down	Aggressive biologic behavior	[39]
	*hMLH1*	Down	Disease progression	[39,40]
	*hPMS1*	Down	Aggressive biologic behavior	[39]
HR	*RAD50*	Up	Higher HR/R activity, mutations, drug resistance	[41]
	*RAD51*	Up	Higher HR/R activity, mutations, drug resistance, progression	[41,42]
	*BRCA1*	SNPs	Therapy outcome	[42,43]
	*RAPR1*	Up/SNPs	Reduced survival, therapy outcome	[42,43]
	*MUTYH*	SNPs	Disease progression	[42]
	*OGG1*	SNPs	Disease progression	[42]
	*PCNA*	SNPs	Disease progression	[42]
	*TPMT*	SNPs	Disease progression	[42]
	*XPC*	SNPs	Disease progression	[42]
NHEJ, alt-NHEJ	*XRCC4*	SNPs	Risk of developing MM	[44,45]
	*XRCC5*	Up/SNPs	OS, risk of developing MM	[45,46]
	*LIG4*	SNPs	Risk of developing MM	[47]
	*DCLRE1C/Artemis*	Up	OS, Risk of developing MM	[46]
	*NSD2*	Up/Down	Drug resistance, accumulation of DNA damage	[48,49]
	*LIG3*	Up	Reduced survival in advance stages, drug resistance	[50]
FA	*FANCF*	Depletion	Overcome resistance	[51]

Up: upregulated, Down: downregulated, OS: overall survival, pts: patients, ASCT: autologous stem cell transplantation, MM: multiple myeloma, NDMM: newly diagnosed MM.

**Table 2:** MAPK inhibitors in MM therapy.

MAPK Pathway Inhibitors		Clinical Trial	Regimes	Clinical Outcome	Ref.
**MEKi**	Selumetinib	NCT01085214	Mono	Low response in RRMM	[105]
	Trametinib	-	Mono	Better response in MAPK-activated pts.	[103]
	Cobimetinib	NCT03312530	Mono	Moderate	[106,107]
			+ venetoclax or + venetoclax with atezolizumab	Higher activity in t(11;14)	
**BRAFi**	Vemurafenib	-	Mono	Rapid response in 1 pt., RRMM with BRAF V600E	[108]
**BRAFi + MEKi**	Vemurafenib + cobimetinib	-	Combo	Promising in relapse MM pts. with BRAF V600E	[109,110]
	Encorafenib + binimetinib	NCT02834364	Combo	Highly effective in RRMM pts. with BRAF V600E/K	[111,112]
	RO-5126766 (also known as CH5126766 and VS-6766)	NCT02407509	Mono	Activity in MM pts. with MAPK pathway mutations	[113]
**RAFi**	Sorafenib	-	+ rapamycin	Anti-MM actions in vitro	[114]
	Sorafenib	NCT00303797	+ bortezomib	Preliminary efficacy	[115]
	Sorafenib	NCT00253578	Mono	Partial response in 2/11 pts. in RRMM	[116]
**p38i**	Talmapimod	NCT00087867	Mono or + bortezomib	Encouraging response in RRMM	[117]
	Plitidepsin	NCT00229203	Mono	13% response in RRMM	[118]
			+ dexamethasone	Up to 22% response in RRMM	

Mono: monotherapy, Combo: combined, RRMM: relapsed/refractory multiple myeloma, MM: multiple myeloma, pts.: patients.

## Data Availability

The data presented in this study are openly available in the reference section.
